# New Method Based on the Direct Analysis in Real Time Coupled with Time-of-Flight Mass Spectrometry to Investigate the Thermal Depolymerization of Poly(methyl methacrylate)

**DOI:** 10.3390/polym15030599

**Published:** 2023-01-24

**Authors:** Rana Salem Al Khulaifi, Mohammed Mousa AlShehri, Ahmad Abdulaziz Al-Owais, Tahani Saad Algarni, Waseem Sharaf Saeed, Ahmed Yacine Badjah-Hadj-Ahmed, Taieb Aouak

**Affiliations:** Department of Chemistry, College of Science, King Saud University, Riyadh 11451, Saudi Arabia

**Keywords:** poly(methyl methacrylate), isothermal decomposition, direct analysis in real time coupled with time-of-flight mass spectrometry

## Abstract

In this work, the isothermal decomposition of poly(methyl methacrylate) synthesized in bulk by the radical route of methyl methacrylate in the presence of azobisisobutyronitrile as the initiator was carried out and monitored for the first time with the DART-Tof-MS technique at different temperatures. Nuclear magnetic resonance (NMR) analysis revealed a predominantly atactic microstructure, and size-exclusion chromatography (SEC) analysis indicated a number average molecular weight of 3 × 10^5^ g·mol^−1^ and a polydispersity index of 2.47 for this polymer. Non-isothermal decomposition of this polymer carried out with thermogravimetry analysis (TGA) showed that the weight loss process occurs in two steps. The first one starts at approximately 224 °C and the second at 320 °C. The isothermal decomposition of this polymer carried out and monitored with the DART-Tof-MS method revealed only one stage of weight loss in this process, which begins at approximately 250 °C, not far from that of the second step observed in the case of the non-isothermal process conducted with the TGA method. The results obtained with the MS part of this technique revealed that the isothermal decomposition of this polymer regenerates a significant part of methyl methacrylate monomer, which increases with temperature. This process involves radical chain reactions leading to homolytic chain scissions and leading to the formation of secondary and tertiary alkyl radicals, mainly regenerating methyl methacrylate monomer through an unzipping rearrangement. Although they are in the minority, other fragments, such as the isomers of 2-methyl carboxyl, 4-methyl, penta-2,4-diene and dimethyl carbate, are also among the products detected. At 200 °C, no trace of monomer was observed, which coincides with the first step of the weight loss observed in the TGA. These compounds are different to those reported by other researchers using TGA coupled with mass spectrometry in which methyl isobutyrate, traces of methyl pyruvate and 2,3-butanonedione were detected.

## 1. Introduction

Poly(methyl methacrylate) (PMMA) is an essential material in the industrial field. This polymer is the source of several applications, such as optic fibers for the transmission of light, plastic glasses, contact lenses, materials for dental prostheses, signs and displays, and billboard liquid crystal displays (LCDs) [1,2,3,4]. The wide applicability of this polymer in different sectors of industry is due to its exceptional properties, such as transparency, toughness, glass-like refractive index, non-toxicity and resistance to impact and to most chemicals. Moreover, its production is expected to increase over the next few years and reach 8.16 billion USD by 2025. This will put pressure on its average price, which is already high compared to other traditional polymers, and lead to growing concerns about the waste management of this material [5,6].

The early studies of the bulk thermal depolymerization of PMMA date from the end of the 1940s. Indeed, several researchers [7,8,9,10] at the time investigated this important aspect of polymers in order to understand the mechanisms involved in such a process. The thermal depolymerization of PMMA was also carried out in solution in diphenyl ether, 1,2,4-trichlorobenzene and in α-methylnaphthalene. Some experiments conducted by Bywater and Black [9] suggested that the mechanism of this degradation involved end-of-chain initiation, de-propagation and chain breaking by solvent transfer. These processes were observed in all the studied solvents. The degradation by chain transfer was increasingly efficient in the trichlorobenzene < diphenylether < α-methylnaphthalene series, while the chain initiation rate was almost the same in all solvents. Afterwards, several works on this subject have been achieved.

The decomposition of PMMA in all its microstructures has been widely investigated with thermogravimetry analysis (TGA). According to different studies, it has been reported that the non-isothermal decomposition of this polymer depends mainly on its tacticity, its molecular weight and the initiator involved in the polymerization. Indeed, Jellinek and Luh [11] investigated the thermal decomposition of isotactic and syndiotactic PMMA with TGA in a closed system over a range of temperatures comprised between 300 °C and 400 °C in an inert atmosphere. These authors reported that the initial string lengths of the isotactic polymers were approximately ten- to twenty-times longer than those of the syndiotactic microstructures. The depolymerization characteristics of the isotactic polymers showed a kinetic chain length less than that of the polymer chains, whereas the depolymerization characteristics of the syndiotactic microstructures exhibited a kinetic chain length of the same order of magnitude as the length of the polymer chain.

Regarding the effect of molecular weight on the decomposition of this polymer, Ferriol et al. [12] investigated the degradation of two PMMA samples synthesized by a free radical polymerization route that had molecular masses of 350,000 and 996,000 g·mol^−1^. The obtained TGA thermogram showed three weight-loss stages for the PMMA with 3.5 × 10^5^ g·mol^−1^, while its DTG curve revealed four steps of decomposition. A similar behavior was also reported by Manring et al. [13]. In the same area, Chen [14] reported a study on the depolymerization of PMMA with different molecular masses in toluene, ethylacetate and chloroform; the obtained results revealed that the polymer with 2.2 × 10^5^ g·mol^−1^ underwent degradation, while the low molecular weight sample showed a depolymerization process. Kashiwagi et al. [15] established that the first thermal decomposition of PMMA prepared by a free radical route takes place in three steps. The least stable step (approximately 165 °C) would be initiated by head-to-head (H–H) bond scissions with the H–H bond dissociation energy estimated to be lower than that of a backbone bond C–C due to a large steric hindrance and the inductive effect of the vicinal ester groups. The second step, which occurs at approximately 270 °C, corresponds to the chain scission of the terminal unsaturated groups (resulting from termination by disproportionation) involving homolytic β scission to the vinyl group. The last step (approximately 350 °C) is initiated by a random scission within the polymer chain.

The effect of terminal groups or the nature of the initiator used in the preparation of PMMA on its thermal decomposition has been studied by Li and Ren [16]. They revealed that, when a thiol was used as an initiator, the thermal degradation of the resulting polymer mainly led to the monomer. The mechanism suggested by these authors involved only the scission of the main chains. Grassie et al. [17] studied the non-isothermal decomposition of polydisperse PMMA with molecular weights that ranged between 3.6 × 10^4^ and 1.79 × 10^5^ g·mol^−1^ obtained by a free radical polymerization route using different initiators. They concluded that the nature of the terminal group had an effect on the rate of degradation, such that the introduction of 1,4-diamino anthraquinone terminal groups prevented the degradation of PMMA at 220 °C. Similar results were also described by Madorsky [18] who found that PMMA initiated with benzoyl peroxide was thermally much less stable than that thermally polymerized in the absence of an initiator. PMMA-benzoyl peroxide started to degrade slowly at 240 °C, while thermally polymerized PMMA degraded at a similar rate at 310 °C. Brockhaus and Jenckel [19] investigated the effect of molecular weight on the depolymerization of PMMA prepared with free radical polymerization initiated with benzoyl peroxide at temperatures in the range between 250 and 350 °C. These authors deduced that, during the decomposition process, two reactions occurred: one initiated at the unsaturated and the other at the saturated chain ends.

The non-isothermal decomposition of PMMA with 9.96 × 10^5^ g·mol^−1^ synthesized with free radical polymerization was studied by Peterson et al. [20] using TGA. The weight loss observed was found to occur stepwise, beginning at 150 °C before slowing down momentarily at 300 °C (with 40 weight% loss) and continuing in a second degradation step. These results seem to be consistent with those obtained by Grassie [17] and Madorsky [18] for PMMA initiated with benzoyl peroxide. The two-step decomposition process of this polymer does not agree with the results obtained by Holland and Hay [21] in the non-isothermal mode of synthesized commercial PMMA involving free radicals, which generated a single decomposition in one step starting at 290 °C. Recently, Godiya et al. [22] used thermogravimetric analysis coupled with mass spectrometry (TGA-MS) to study the depolymerization of PMMA. The results obtained revealed that, in this process, the polymer chains were decomposed mainly into methyl methacrylate and also into a few non-polymerizable species that prevented the re-polymerization of the recovered monomer. This study stated that, besides the main by-product (methyl isobutyrate), traces of methyl pyruvate and 2,3-butanonedione were also formed during the thermal depolymerization of PMMA. The 2,3-butanedione formed was found to be responsible for the unpleasant odor in the recovered MMA.

To optimize the recovery of methyl methacrylate (MMA) resulting from the depolymerization of polymethyl methacrylate (PMMA) dental resin fragments/residues in order to pilot the experiments at technical scale, Bisi dos Santos et al. [23] used the thermogravimetric analysis (TG/DTG/DTA). The liquid-phase products obtained at 420 °C were subjected to fractional distillation. The results revealed that the depolymerization of PMMA dental resin waste led to methyl methacrylate with concentrations varying between 83.454 and 98.975%. This study also showed that the optimum operating conditions to achieve high MMA concentrations, as well as elevated yields of liquid reaction products, were 345 °C and 80 min. The influence of temperature on the recovery and purity of methyl methacrylate (MMA) by depolymerization of polymethyl methacrylate (PMMA) dental resin scraps was investigated by Ferreira et al. [24]. The GC–MS analysis identified methyl methacrylate (MMA) and ethylene glycol dimethacrylate (EGDMA). Methyl methacrylate concentrations varied between 94.20 and 95.66%, showing a moderate increase with rising depolymerization temperature.

Through our literature review, we did not find any detailed articles dealing with the isothermal decomposition of PMMA and the products resulting from this process. In the present investigation, our objective was to study the isothermal decomposition of PMMA using, for the first time, a new technique based on direct analysis in real-time coupled with time-of-flight mass spectrometry (DART-ToF-MS), the results being compared with those obtained with a non-isothermal process carried out with TGA. To achieve this goal, PMMA was synthesized by the bulk polymerization of methyl methacrylate using AIBN as the initiator. The structure and tacticity of the prepared polymer were characterized with nuclear magnetic resonance (NMR), and the average molecular weights were determined with size-exclusion chromatography (SEC). The isothermal decomposition of the synthesized polymer was investigated with DART-ToF-MS at temperatures ranging between 200 and 550 °C, while the non-isothermal decomposition of this same polymer was carried out with the TGA method from 25 to 600 °C. DSC analysis was also employed in this work to characterize the different transitions of the synthesized PMMA, notably that of the first mass loss observed on the TGA thermogram cited in the literature between 150 and 270 °C. The main products or their isomers generated from the isothermal decomposition process of PMMA were elucidated in the MS part of the present study.

## 2. Materials and Methods

### 2.1. Materials

Methyl methacrylate (MMA) (purity, ≥99%), azobisisobutyronitrile (AIBN) (purity, ≥98%), anhydrous tetrahydrofuran (THF) (purity, ≥99.9%) and *n*-heptane (purity, 99%) were provided from Sigma Aldrich (Germany). The monomer was purified by distillation under reduced pressure and stored before use under nitrogen gas. The initiator was purified three times by recrystallization in absolute ethanol. The solvents and precipitants were used without further purification.

### 2.2. Preparation of Poly(methyl methacrylate)

PMMA was prepared by a free radical bulk polymerization route of MMA in the presence of AIBN as the initiator at 70 °C. A total of 30 g (0.3 mol) of MMA was introduced in a three-necks bottom flask containing 11.5 mg (0.07 mmol) of AIBN linked to a refrigerant by the main opening. The cooler was connected at its top opening to a bubbler containing silicone oil. Through one of the two secondary openings was introduced the monomer, and through the other, nitrogen gas flowed at a rate of 3 mL·min^−1^, which produced bubbles that expelled the air from the reactor and ensured the homogenization of the reaction mixture and the temperature of the reaction. A very viscous solution was obtained after approximately 20 min of the reaction. The reactor was then allowed to cool in air to the ambient temperature (~25 °C), and the polymer obtained was then isolated by precipitation in *n*-heptane. To eliminate the residual monomer and oligomers, the PMMA collected was purified three times by dissolution in THF and then precipitated in heptane. The polymer was dried in open air for 12 h and then under vacuum at 50 °C for 24 h. The average percentage yield of the polymerization was 75.22 wt%. This procedure was triplicated three times in the same conditions, and the results of the polymerization were taken from the arithmetic mean of the three trials. The apparent molecular weight, *M*, estimated from the percentage yield of the polymerization and Equation (1) [25] was 1.61 × 10^5^ g·mol^−1^.

(1) $$M=\frac{yield\times n_{MMA}\times M_{MMA}}{2\times 100\;\times n_{AIBN}}$$

where $n_{MMA}$ and $n_{AIBN}$ are the mole numbers of MMA and AIBN, respectively, $M_{MMA}$ is the molar mass of MMA and 2 is the number of free radicals that resulted from the dissociation of AIBN.

### 2.3. Characterization of Equipment/Instruments

#### 2.3.1. NMR Analysis

The structure and the tacticity of the polymer obtained were examined with ^1^HNMR et ^13^C NMR in CDCl_3_ at room temperature (~25 °C) using a JEOL FX 90 Q NMR apparatus at 500 and 200 MHz, respectively.

#### 2.3.2. SEC Analysis

The average number molecular weights of the prepared PMMA were estimated in THF at 30 °C with size-exclusion chromatography (SEC) on a Varian apparatus. This instrument is equipped with a JASCO-type 880-PU HPLC pump with a flow rate of 1.0 mL, refractive index and UV detectors and Shodex GPC KF-806 M (8.0 mm I.D. × 300 mm) columns calibrated with polystyrene standards; the results obtained indicated 2.30 × 10^5^ g·mL^−1^, 5.67 × 10^5^ g·mL^−1^ and 2.47 for ${\bar{M}}_{n}$, ${\bar{M}}_{w}$ and polydispersity index (I), respectively. The average polymerization degree ($\bar{D}p)$ of the prepared PMMA, which is by definition equal to the average molecular weight of the polymer divided by the molar mass of the monomer unit, calculated from Equation (2) [26] was 2297.

(2) $$\bar{D}p=\frac{\bar{M}n}{M_{MMA}},$$

#### 2.3.3. TGA Analysis

The TGA of PMMA was performed under dynamic nitrogen gas on a TGA/DSC Mettler-Toledo thermogravimeter (Columbus, OH, USA). Samples weighing between 10 and 14 mg were loaded into the TGA aluminum pan and then heated from 25 °C to 600 °C at a heating rate of 50 °C·min^−1^.

#### 2.3.4. DSC Analysis

The DSC thermogram of PMMA was traced using a DSC device (Shimadsu DSC 60, Kyoto, Japan) previously calibrated with indium. Between 10 and 12 mg of the samples were packed in aluminum DSC pans before being placed in the DSC cell. The samples were scanned by heating from 30 to 280 °C with a heating rate of 10 °C·min^−1^. The value of the glass transition temperature (Tg) was taken from the inflection point of the thermal curves.

#### 2.3.5. DART-ToF-MS Analysis

The isothermal decomposition of PMMA was analyzed with an Accu-ToF LC-plus JMS-T100 LP mass spectrometer JEOL (Tokyo, Japan) working with a direct-analysis-in-real-time (DART) ion source (Ion Sense, Saugus, MA, USA). The sample was used without any prior preparation. The fragments that resulted from the decomposition process were evaporated in a stream of helium atmosphere heated at constant temperatures of 200, 250, 300, 350, 400, 450, 500 and 550 °C. The heated helium/vapor mixture was then ionized by excited metastable helium atoms before entering the ion source of the time-of-flight mass spectrometer. All the samples were analyzed with DART-ToF-MS using the Accu-TOF mass spectrometer acquired from JEOL (Tokyo, Japan). The experimental conditions were as follows: vacuum level 1.3 × 10^5^ Pa, He used as a heating and ionization gas, ring lens with a voltage of 4 V, peaks voltage of 500 V, and a mass resolution that ranged between 3600 and 4900. The sample (1–2 mg) in powder form was placed in a small piece of sandpaper and introduced between the DART outlet and the MS port inlet, and polyethylene glycol (10^4^ g·mol^−1^) was used in this work for calibration.

## 3. Results and Discussion

The characterization of PMMA was carried out on the three replicas, and the data obtained were taken from their arithmetic means.

### 3.1. NMR Analysis

**Figure 1:**
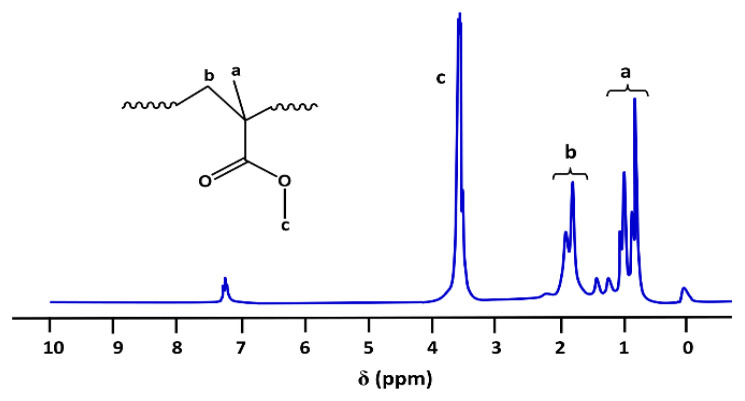
^1^HNMR spectrum of PMMA obtained in CDCl_3_.

**Figure 2:**
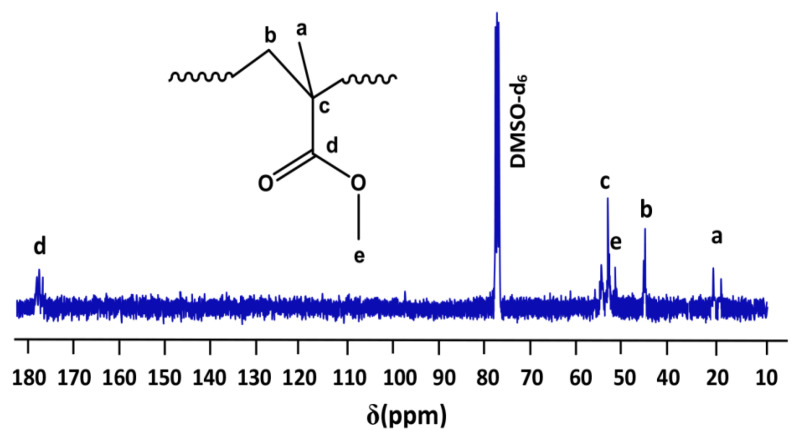
^13^CNMR spectrum of the prepared PMMA.

The structure and the microstructure of the synthesized PMMA were characterized with ^1^H and ^13^CNMR, and Figure 1 and Figure 2 show the different signals of protons and carbon-13, respectively. As can be seen from the ^1^HNMR spectrum, the characteristic peaks centered at 0.92, 1.78 and 3.51 ppm are attributed to the protons of methyl (a), ethyl (b) and methyl (c) groups, respectively. No other significant peaks assigned to impurities or residual monomers are observed in this spectrum.

The ^13^C NMR spectrum of the prepared PMMA in Figure 2 shows the structure of this polymer through the five signals attributed to the carbons a, b, c, d and e at 20.0, 45.5, 51.3, 53.0 and 177.6 ppm, respectively.

**Figure 3:**
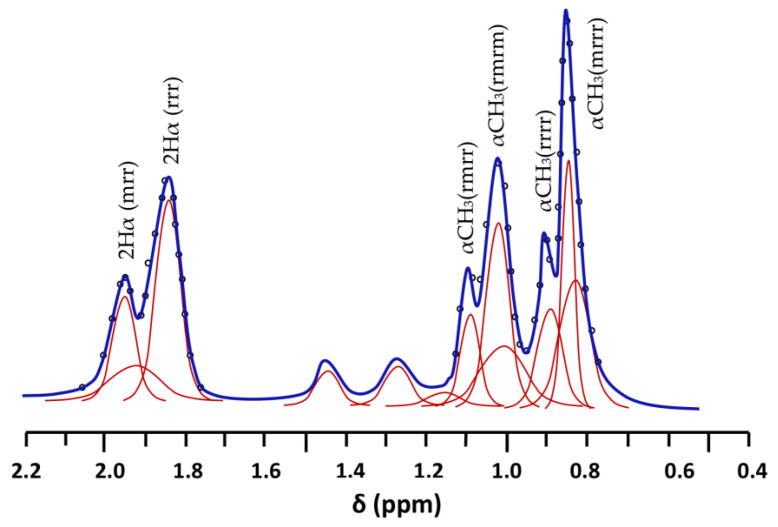
Deconvoluted ^1^HNMR spectrum of PMMA in Laurentzian curves between 0.40 ppm and 2.20 ppm.

The tacticity of PMMA was estimated from the deconvolution of the localized overlapping signals in the ^1^HNMR spectrum between 0.62 and 1.63 ppm assigned to the methyl groups (a) belonging to different tetrads. According to the literature [27], the tetrads characterizing the tacticities of PMMA are gathered in Table 1. The deconvolution of these overlapping signals in Laurentzian peaks led to the results in Figure 3. The rate of each triad composing the polymer sample is calculated from the following equation:

(3) $$Tetrad_{i}\left(\%\right)=\frac{a_{i}}{a_{T}}\times 100$$

were $a_{i}$ and $a_{T}$ are the surface area of the triad i and the total area, respectively.

**Figure 4:**
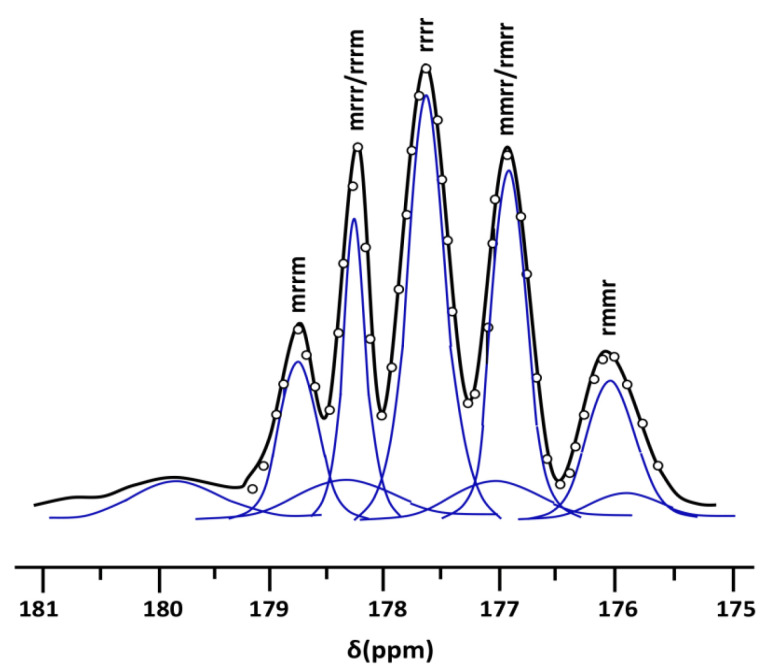
Deconvoluted ^13^CNMR spectrum of PMMA in Laurentzian curves between 175 ppm and 181 ppm.

On the other hand, the deconvolution of the signals of the carbon (d) in the ^13^CNMR spectrum in Figure 2 that appear between 176 and 180 ppm, as shown in Figure 4, reveals the presence of signals belonging to the microstructures of the mrrm, mrrr/rrrm, rrrr, mmrr/rmrr and rmmr tetrads at 178.75, 178.27, 177.80, 176.90 and 176.05 ppm, respectively [27]. The integration of these signals made it possible to estimate the tacticity of this polymer, and the results obtained are grouped with those determined with ^1^HMR in Table 1.

### 3.2. TG-Analysis

**Figure 5:**
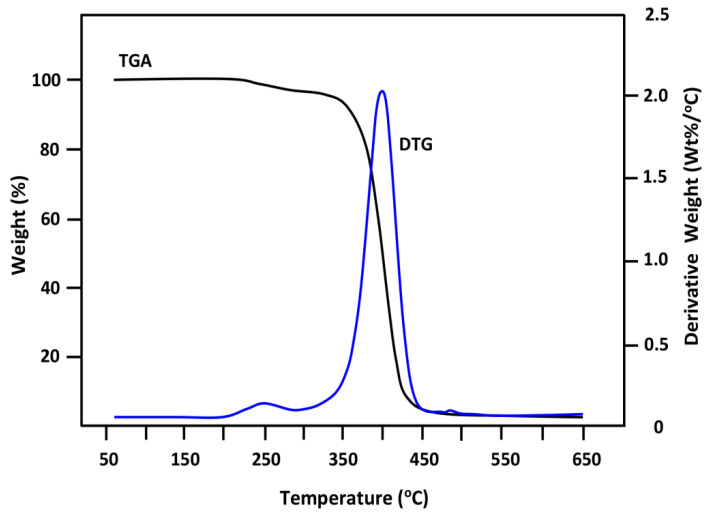
Thermograms TGA/DTG of the prepared PMMA.

The thermogram TGA/ATD of the synthesized PMMA (${\bar{M}}_{n}=2.3\times 10^{5}$ g·mL^−1^, ${\bar{M}}_{w}/{\bar{M}}_{n}=2.47$), with a microstructure that is mainly atactic, is shown in Figure 5. This thermal curve shows two stages of the weight loss in its decomposition process. The first step begins at approximately 224 ± 3 °C and ends at 272 ± 3 °C in which 4.5 ± 0.6 wt% of the sample is volatilized, and the second step at 320 ± 4 °C in which a large amount of monomer is recovered. According to certain authors [16,28], during the first step, the small amount of the sample released in the first step contains residual non-reacted monomer, solvent and/or precipitant encrusted in the polymer. Indeed, the effect of the residual solvent involved in the synthesis of this polymer on its thermal decomposition behavior was investigated by Kizilduman et al. [29]. It was found that the first weight loss begins at 288 °C (acetone), 152 °C (THF) and 154 °C (chloroform and toluene). Other authors attribute this first step to the thermal decomposition of PMMA initiated by scissions of the head-to-head linkages (H–H) [15,30,31]. The second stage of PMMA obtained in this work involves a radical decomposition process, which begins from the end of the chains containing unsaturated bonds, regenerating a large amount of monomer resulted by the depolymerization reaction [22].

### 3.3. DSC Analysis

The DSC analysis of the prepared PMMA aims to remove the nuance on the character of the weight loss observed at approximately 200 °C in the TGA/TDA thermograms of this polymer.

**Figure 6:**
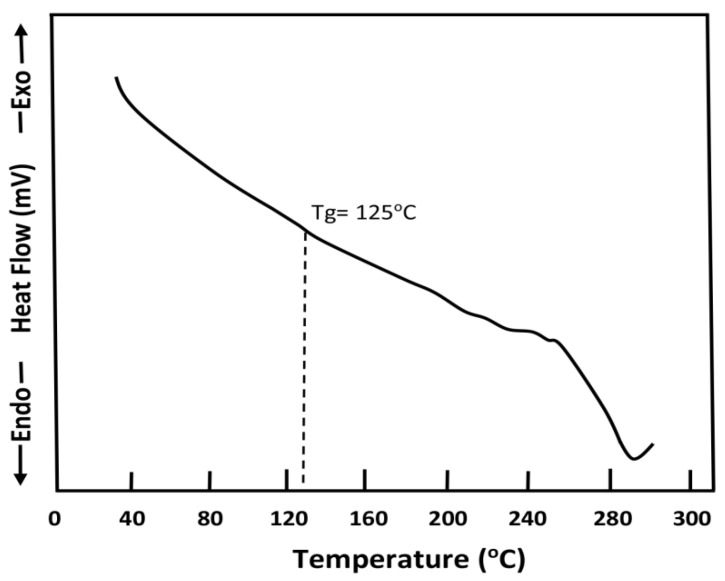
DSC thermogram of the prepared PMMA.

Indeed, the profile of the DSC thermogram in Figure 6 shows the glass transition temperature for this PMMA at 125 ± 2 °C, which agrees with the literature [32]. The small endothermic peaks localized between 188 ± 2 °C and 288 ± 2 °C, which coincide with the first temperature of mass loss observed on the TGA thermogram, are probably attributed to the vaporization temperatures of some fragments released from the first step of the decomposition of this polymer. Such a thermal behavior was also observed on the DSC thermograms of PMMA by Ulu et al. [31] and El-Zaher et al. [33] and also attributed to the decomposition of this polymer.

### 3.4. DART-ToF-MS Study

**Figure 7:**
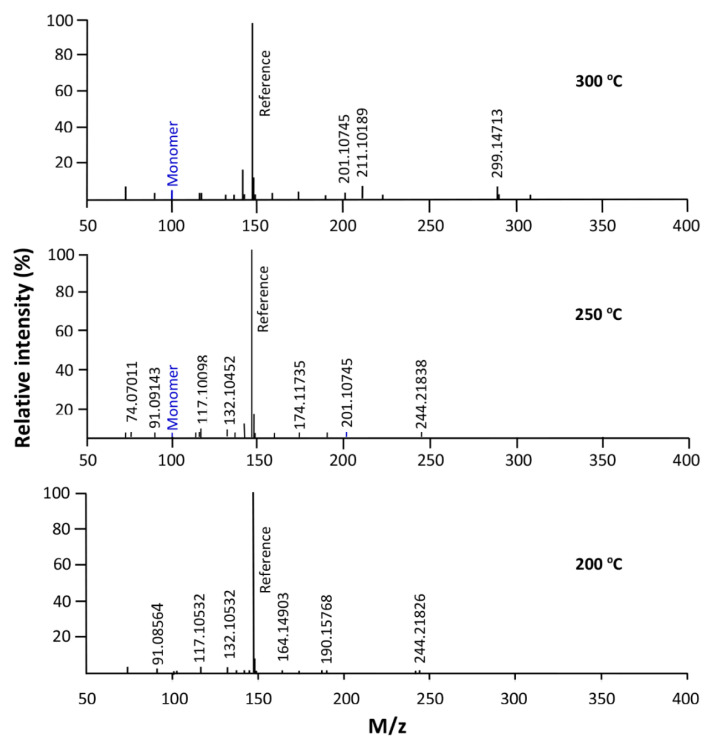
DART-Tof-MS spectrum of the isothermal decomposition of PMMA obtained in gas nitrogen at 200, 250 and 300 °C.

**Figure 8:**
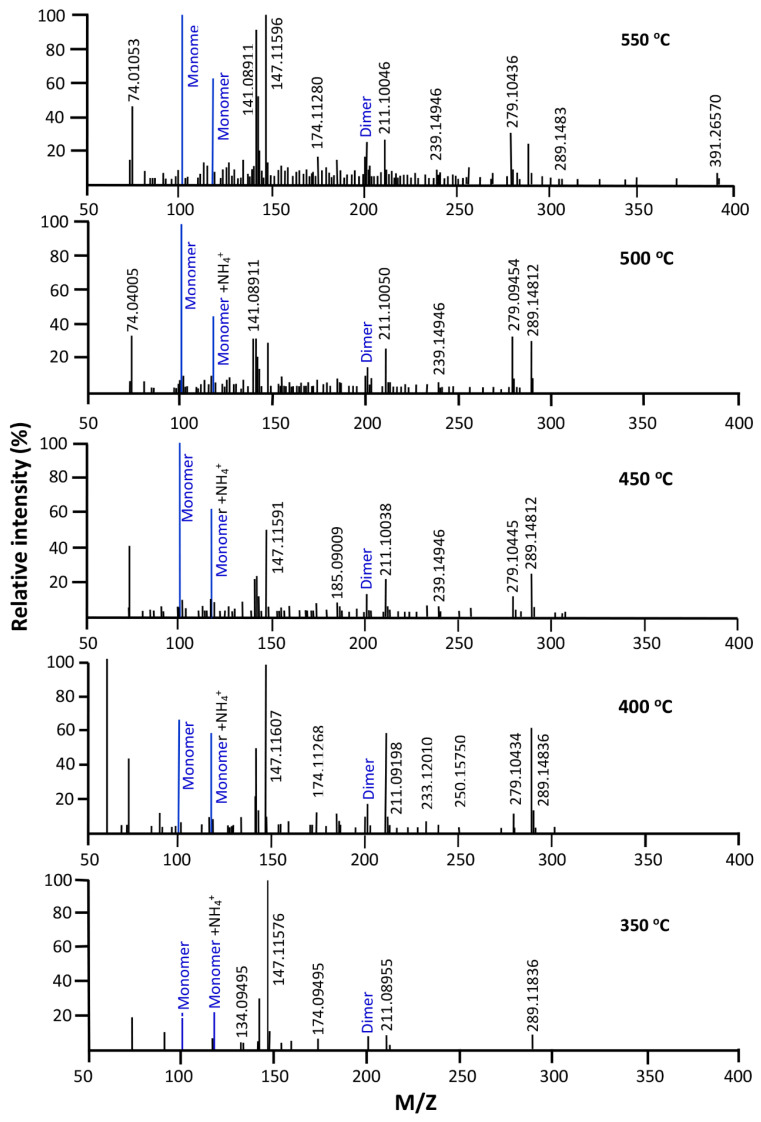
DART-Tof-MS spectrum of the isothermal decomposition of PMMA obtained in gas nitrogen at 350, 400, 450, 500 and 550 °C.

For the characterization of the polymer samples, the experimental parameters used in the DART-ToF-MS were optimized. The best results were observed in the positive ionization mode. Most of the mass spectral peaks corresponded to protonated ions [M + H]^+^, while the molecular peaks M^+^ were also present for a few compounds. Also, in some cases, the MS spectra showed the simultaneous presence of adduct ions [M + NH_4_]^+^ resulting from the addition of an atmospheric ammonium ion to the molecule. The isothermal decomposition of PMMA performed by means of DART-ToF-MS in a stream of helium atmosphere was carried out at temperatures that ranged between 200 and 550 °C, and the results obtained are shown in Figure 7 (200, 250 and 300 °C) and Figure 8 (350, 350, 400, 450, 500 and 550 °C). The characteristics of the fragments that resulted from this decomposition are grouped in Table 2. As can be observed from these data, PMMA begins its thermal decomposition at approximately 200 °C. This value is not very far from that observed on the thermogram of the TGA (224 °C), although the heating mode used in this method, which is isothermal, is different to that of TGA, which is non-isothermal.

At 200 °C, in addition to the intense signal of cyclopentyl benzene used in this study as an internal reference that appears on the DART-Tof-MS spectrogram at 147.116 *m*/*z*, other small signals are observed, the most important of which appear at 91.085, 117.105, 132.105 and 141.097 *m*/*z*, which do not characterize the monomer, and their intensities increase with temperature. This confirms the presence of the endothermic peaks observed in the DSC thermogram between 188 and 288 °C, indicating the vaporization of the released molecules with molar masses varying between 91 and 141 g·mol^−1^, such as the isomers of leucine (C_6_H_14_NO_2_), 5-amino-5-oxo-1-pentanaminium(C_5_H_13_N_2_O) and 2-Methyl carboxyl, 4-methyl, penta-2,4-diene (C_8_H_13_O_2)_. This coincides well with the temperature of the first stage of mass loss at 224 °C already observed on the TGA thermogram. These results remove the nuance on the nature of the released products during the first step of the mass loss in which certain authors using the TGA method [16,28] attribute them to the residues of monomer and traces of solvent and precipitant incrusted in the polymer matrix.

**Scheme 1:**
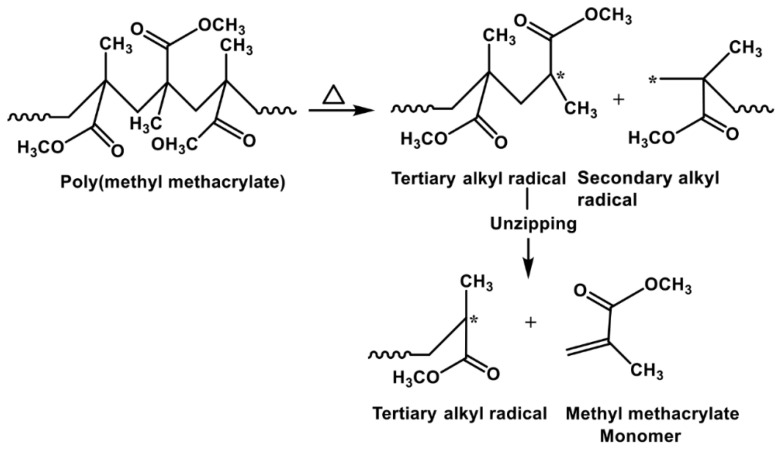
Probable mechanism of the depolymerization of PMMA.

At 350 °C and more, the thermal decomposition of PMMA regenerates a significant amount of monomer, which increases with temperature. This finding confirms the results reported by different authors using the thermal pyrolysis [3,34,35,36] and the TGA methods [18,19]. The mechanism of the thermal depolymerization of PMMA in an inert medium is well known in the literature [28,37,38,39,40]. According to these authors, the thermal decomposition of PMMA involves radical chain reactions leading to homolytic scissions of the chains and leading to the formation of secondary and tertiary alkyl radicals, mainly regenerating methyl methacrylate monomer through an unzipping rearrangement as shown in Scheme 1. What still remains random and poorly known is the mechanism highlighted to obtain the other resulting fragments due to the uncontrollable size and movement of the radicals formed in the degradation process. For this reason, the products resulted from the decomposition of this polymer other than MMA differ from one method used to another and one experiment to another. For example, Zeng et al. [28], using TGA coupled with MS (TGA-MS) to study the non-thermal decomposition of PMMA, revealed a large amount of monomer and other byproducts, such as methyl isobutyrate, traces of methyl pyruvate and 2,3-butanedione, which are totally absent in the DART-ToF-MS thermograms.

The chemical formulas and the saturation degrees of the fragments that resulted from this decomposition and were estimated through the data of the MS part of this technique are gathered in Table 2, and the proposed names and structures of the main fragments and/or their isomers are grouped in Table 3. Except for the regeneration of the monomer obtained as indicated by Scheme 1, the other molecules are resulted from a random recombination of different radicals resulting from the thermal decomposition of PMMA.

## 4. Conclusions

DART mass spectrometry was successfully applied to study the isothermal decomposition of PMMA. It was found that the non-isothermal decomposition of PMMA studied with TGA shows two steps of weight loss. The first one begins at approximately 224 °C, and the second one starts at 320 °C. However, the isothermal decomposition of this polymer carried out with the DART-Tof-MS method revealed only one stage of weight loss, which started at approximately 200 °C. At this temperature and more, the results of the thermal decomposition of PMMA were a regeneration of a significant part of the monomer confirming those of the literature. It was also revealed that the amount of MMA recovered increased with temperature to reach a maximum at 550 °C. Among the molecules released during the first stage of the decomposition process, no trace of monomer was detected, thus eliminating the nuance on the nature of the products generated during this stage.

## Figures and Tables

**Table 1:** ^1^H NMR assignments characterizing the triads and tetrads in PMMA using CDCl_3_ as the solvent [27].

Assignment	δ (ppm)	Assignment	δ (ppm)
1Hα (mmr)	2.11, 2.07	αCH_3_ (rmmr)	1.23 (shoulder)
1Hα (rmr)	2.03, 1.98	αCH_3_ (rmmm)	1.22
2Hα (mrr)	1.93	αCH_3_ (mmmm)	1.21 (shoulder)
2Hα (rrr)	1.85	αCH_3_ (rmrr)	1.08
1Hα (mmr)	1.53, 1.49	αCH_3_ (rmrm)	1.05 (shoulder)
1Hβ (rmr)	1.49, 1.45	αCH_3_ (rrrr)	0.95
-	-	αCH_3_ (mrrr)	0.94

**Table 2:** Fragments resulted from the isothermal decomposition of the PMMA.

N^o^	m ^exp^	m ^calc^	Δm (mmu)	Formula	Unsaturation Degree	Proposed Compound
1	100.05989	100.05243	7.46	C_5_H_8_O_2_	2.0	MMA
2	101.05463	101.06025	−5.62	C_5_H_9_O_2_	1.5	
3	102.06018	102.06361	−3.43	C_4_^13^CH_9_O_2_	1.5	
4	103.06470	103.06696	−2.26	C_3_^13^C_2_H_9_O_2_	1.5	
5	118.08603	118.08680	−0.78	C_5_H_12_NO_2_	0.5	MMA + NH_4_^+^
6	119.08895	119.09016	−1.21	C_4_^13^CH_12_NO_2_	0.5	
7	120.08965	120.09351	−3.86	C_3_^13^C_2_H_12_NO_2_	0.5	
8	141.09754	141.09155	5.99	C_8_H_13_O_2_	2.5	
9	142.09539	142.09491	0.49	C_7_^13^CH_13_O_2_	2.5	
10	147.11596	147.11738	−1.42	C_11_H_15_	4.5	
11	148.11339	148.12073	−7.34	C_10_^13^CH_15_	4.5	
12	174.11280	174.11302	−0.22	C_8_H_16_NO_3_	1.5	C_8_H_12_O_3_ + NH_4_^+^
13	175.11237	175.11637	−4.00	C_7_^13^CH_16_NO_3_	1.5	-
14	200.11071	200.10754	3.17	C_13_H_14_NO	7.5	C_13_H_10_O + NH_4_^+^
15	201.10745	201.11268	−5.24	C_10_H_17_O_4_	2.5	Dimer
16	211.10046	211.09703	3.43	C_11_H_15_O_4_	4.5	
17	279.10436	279.10799	−3.64	C_11_H_19_O_8_	2.5	
18	280.13415	280.13107	3.08	C_15_H_20_O_5_	6.0	
19	289.14834	289.14666	1.68	C_20_H_19_NO	12.0	C_20_H_15_O + NH_4_^+^
20	290.15650	290.15449	2.01	C_20_H_20_NO	11.5	C_20_H_16_O + NH_4_^+^

**Table 3:** The main probable molecules or their isomers resulting from the decomposition of PMMA.

N^o^	Unsat. Deg.	Formula	Name	Structure
1, 2, 3, 4	2	C_5_H_8_O_2_	Methyl methacrylate	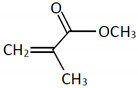
	4	C_7_H_8_O_2_	Methyl 1,3-cyclopentadiene-1-carboxylate	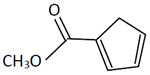
8	3	C_8_H_13_O_2_	2-Methyl carboxyl, 4-methyl, penta-2,4-diene	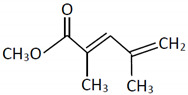
	3	C_10_H_16_O_4_	2,5-Dimethylcarboxyl -5-methyl, pent-3-ene	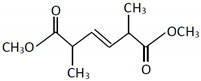
15	4	C_10_H_14_O_4_	2,5-Dimethyl carboxyl, 5-methyl, penta-2,4-diene 2,4	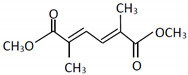
	3	C_10_H_18_O_4_	1,1-Dimethyl, 1,4-dimethylcarboxyl, hex-4-ene	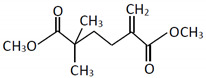
16	4.5	C_11_H_14_O_4_	Dimethyl carbate	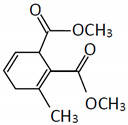
16	4.5	C_11_H_14_O_4_	Dimethyl carbate	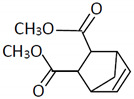
18	6	C_15_H_19_O_5_	Methyl carboxylate-2, penta-2,4-diene, cyclohexan- α-one, β -methylcarbxyl	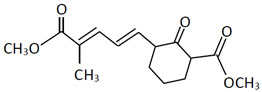

## Data Availability

The data presented in this study are available in the article.
